# β2-microglobulin has a different regulatory molecular mechanism between ER^+^ and ER^−^ breast cancer with HER2^−^

**DOI:** 10.1186/s12885-019-5410-1

**Published:** 2019-03-12

**Authors:** Dandan Chai, Kesheng Li, Huifen Du, Suisheng Yang, Rong Yang, Yang Xu, Xiaowen Lian

**Affiliations:** 1 Department of Medicine Biotechnology, Medicine and Science Research Institute of Gansu Province, Lanzhou, China; 2 Department of Breast Surgery, Tumor Hospital of Gansu Province, Lanzhou, China; 3 Department of Pathology, Tumor Hospital of Gansu Province, Lanzhou, China

**Keywords:** β2M, Regulatory, Molecular, Mechanism, Breast cancer

## Abstract

**Background:**

Previous studies have demonstrated that β2-microglobulin (β2M) promotes the growth and survival of a variety of cancer cells and has different regulatory effects on the expression of Bcl-2 and HER2 in HER2^−^ breast cancer cells. However, β2M-mediated signaling in ER^+^ and ER^−^ breast cancer with HER2^−^ remains unclear.

**Methods:**

β2M expression vector and siRNA were transfected into two types of HER2^−^ breast cancer cells, and the possible relevant signaling molecules were subsequently analyzed by real-time PCR and western blotting. These signaling molecules were also analyzed by real-time PCR and immunohistochemistry (IHC) in two types of HER2^−^ breast cancer tissues, and the associations between β2M and these signaling molecules were assessed using Spearman’s correlation analysis.

**Results:**

β2M silencing downregulated p-SGK1/SGK1 levels and Bcl-2 expression, and β2M overexpression downregulated p-CREB/CREB and significantly upregulated p-SGK1/SGK1 levels and Bcl-2 expression, and both resulting processes did not affect HER2, HIF-1α, VEGF, and ERK signaling in ER^+^ breast cancer cells with HER2^−^. β2M silencing upregulated p-CREB/CREB and VEGF protein and significantly downregulated p-ERK/ERK levels, and β2M overexpression downregulated p-CREB/CREB and VEGF, significantly upregulated p-ERK/ERK levels, and both resulting processes did not affect HIF-1α and SGK1 signaling in ER^−^ breast cancer cells with HER2^−^. β2M expression was positively correlated with p-CREB, p-SGK1, and Bcl-2 expression and had no correlation with HIF-1α, VEGF, and p-ERK1/2, whereas p-SGK1 exhibited a significantly positive correlation with Bcl-2 expression in cancer tissues of patients with luminal A breast cancer, which coincide with the results obtained from the same molecular types of breast cancer cells except CREB signaling. However, β2M expression did not show a significant correlation with HIF-1α, p-CREB, VEGF, p-SGK1, p-ERK1/2, and Bcl-2 expression in cancer tissues of patients with basal-like breast cancer, which was discordant with the results obtained from the same molecular types of breast cancer cells.

**Conclusions:**

β2M has a different molecular regulatory mechanism between ER^+^ and ER^−^ breast cancer with HER2^−^, and it may promote tumor survival through the SGK1/Bcl-2 signaling pathway in ER^+^ breast cancer with HER2^−^ and has no regulatory effects on ER^−^ breast cancer with HER2^−^.

## Background

β2M is a nonglycosylated protein with a molecular weight of 11.8 kDa. Its serum expression levels are elevated in cancer patients, including patients with breast cancer, thyroid cancer, liver cancer, small cell lung cancer, renal cell carcinoma, prostate cancer, and gastrointestinal cancers [1–9]. Previous studies have demonstrated that β2M enhances the growth and survival of cancer cells through the activation of protein kinase A (PKA)/cAMP-responsive element binding protein (CREB)/vascular endothelial growth factor (VEGF) signaling pathway and cell survival signaling, including the phosphatidylinositol 3-kinase (PI3K)/Akt and extracellular signal-regulated kinase (ERK) pathways [10–13]. The β2M/hemochromatosis protein (HFE) complex could induce epithelial to mesenchymal transition (EMT), which promotes bone and soft tissue metastases of human prostate, breast, lung, and renal cancer cells in vivo by activating the iron-responsive hypoxia-inducible factor-1α (HIF-1α) signaling pathway [14], and β2M could promote tumor growth through the activation of the vascular endothelial growth factor receptor-2 (VEGFR-2)/Akt/mammalian target of rapamycin (mTOR) signaling pathway in ovarian cancer [15]. These findings suggest that β2M can promote tumor growth and metastases, including breast cancer, and the β2M-mediated multiple molecular signaling network is extremely complex.

Some studies have shown that metastatic breast cancer is likely associated with a β2M/creatinine ratio > 3.8 [16], and patients with downregulated β2M show significantly improved overall survival compared to patients with lymph node-positive breast cancer with normal β2M levels [17], and β2M overexpression could drive EMT and promote the growth, invasion, and metastasis of human breast cancer cells [14]. In contrast, other studies have demonstrated that the loss of human leukocyte antigen (HLA) class I or β2M expression is associated with poor prognosis in breast cancer patients [18, 19], and β2M could increase the sensitivity of breast cancer cells MCF-7 to doxorubicin, and a decrease or loss of β2M expression by antisense RNA is involved in the acquisition of doxorubicin resistance [20]. These studies have shown that β2M has two opposite kinds of regulatory effects on breast cancer. In addition, human epidermal growth factor receptor 2 (HER2) overexpression could increase the adhesion, migration, invasion, and metastasis of breast cancer cells [21–23]. Our previous study showed that β2M small interfering RNA (siRNA) could significantly inhibit the B-cell lymphoma 2 (Bcl-2) expression, but not the estrogen receptor (ER), progesterone receptor (PR), and HER2 expression in breast cancer cells MCF-7 with ER-positive (ER^+^), PR-positive (PR^+^), and HER2-negative (HER2^−^) status, whereas the β2M siRNA significantly upregulated the Bcl-2 and HER2 expression in breast cancer cells MDA-MB-231 with ER-negative (ER^−^), PR-negative (PR^−^), and HER2^−^ status [3]. Moreover, the ability of β2M to act as a positive or negative cell growth regulator is cell context-dependent [9]. Thus, the role of β2M in breast cancer may be associated with its heterogeneity. Breast cancer is a remarkably heterogeneous disease and can be subtyped as luminal A, luminal B, HER2 overexpression, and basal-like based on the expression of ER, PR, and HER2 [24]. Therefore, we speculate that β2M may mediate diverse signaling pathways and play disparate roles in different types of breast cancers. Our previous study showed that β2M protein expression is positively associated with ER expression and is not associated with HER2 in breast cancer, and β2M has different regulatory effects on the expression of Bcl-2 and HER2 between ER^+^ and ER^−^ breast cancer cells with HER2^−^ [3]. Therefore, in this study, we investigated whether β2M plays disparate regulatory roles between ER^+^ and ER^−^ breast cancer with HER2^−^.

## Methods

### Cell culture

The human breast cancer cell lines MCF-7 (ER^+^ PR^+^ HER2^−^), T47D (ER^+^ PR^+^ HER2^−^), MDA-MB-231 (ER^−^ PR^−^ HER2^−^), and Hs578T (ER^−^ PR^−^ HER2^−^) were obtained from the Cell Bank of the Chinese Academy of Sciences (Shanghai, China) and cultured in high glucose Dulbecco’s modified Eagle’s medium (DMEM, Thermo Fisher Scientific, Shanghai, China) with 10% heat-inactivated fetal bovine serum (FBS, Minhai Biotechnology Co., Ltd., Beijing, China) at 37 °C with 5% CO_2_.

### Tissue samples

All of the tissue samples in this study were from patients who underwent surgery for breast cancer between 2011 and 2015 and diagnosed by clinical and histopathological evidence at Tumor Hospital of Gansu Province. All patients gave written informed consent. Formalin-fixed, paraffin-embedded tumor and matched adjacent tissues (81 luminal A subtype and 40 basal-like subtype) were obtained from the Department of Pathology, and serial 4-μm sections were processed for the IHC. The tumor and adjacent fresh tissues (29 luminal A subtype and 9 basal-like subtype) were obtained from surgical specimens resected from patients without chemotherapy and radiotherapy before operation, immediately frozen in liquid nitrogen and stored at − 80 °C for preparation of total RNA.

### Silencing of *β2M* gene by siRNA in ER^+^ HER2^−^ and ER^−^ HER2^−^ breast cancer cells

The sequence-specific β2M siRNA (siβ2M) and scrambled siRNA were purchased from GenePharma Co., Ltd. (Shanghai, China). The sequences of the siRNAs are shown in Table 1. Knockdown of β2M expression was achieved using siβ2M, and scrambled siRNA was used as control. siRNA transfection was performed using a Lipofectamine RNAiMAX reagent (Thermo Fisher Scientific) according to the manufacturer’s protocols. Briefly, cells grown on six-well plates were transfected using 40 nM siRNA and 7.5 μL Lipofectamine RNAIMAX per well, and the medium was changed after 6 h. At approximately 48 h post-transfection, the cells were lysed and analyzed using real-time PCR and western blotting.

**Table 1:** Sequences of siRNAs targeting β2M

Name	Sequence
β2M siRNA	Sense, 5′-CACAGCCCAAGAUAGUUAATT-3’
	Antisense, 5′-UUAACUAUCUUGGGCUGUGTT-3’
scrambled siRNA (control)	Sense, 5′-UUCUCCGAACGUGUCACGUTT-3’
	Antisense, 5′-ACGUGACACGUUCGGAGAATT-3’

### Overexpression of β2M in ER^+^ HER2^−^ and ER^−^ HER2^−^ breast cancer cells

To construct the β2M expression vector, β2M cDNA was isolated by reverse transcription-PCR (RT-PCR) from cells and flanked with *Kpn*Iand *Bam*HIcloning sites. The β2M primer sequences were as follows: forward, 5′-CGGGGTACCATGTCTCGCTCCGTGGCCTT-3′; reverse, 5′-CGCGGATCCTTACATGTCTCGATCCCACT-3′. The β2M cDNA was inserted between the *Kpn*Iand *Bam*HIsites in the pEGFP-C1 vector. The recombinant vector was designated pEGFP-C1-β2M, which was subsequently extracted with an E.Z.N.A. Endo-Free Plasmid Mini Kit (Omega Bio-Tek, Norcross, GA, USA). Plasmid concentration was measured using a NanoDrop spectrophotometer (Thermo Fisher Scientific) at a wavelength of 260 nm, and purity was checked by measuring the 260/280 and 260/230 ratios. Transfection was conducted using Lipofectamine 3000 (Thermo Fisher Scientific) as recommended by the manufacturer. Briefly, cells were seeded in six-well plates at a density of 1.5–3 × 10^5^ per well. After 24 h, cells were transfected using 2.5 μg of pEGFP-C1-β2M and 3.75 μL Lipofectamine 3000 per well, medium was changed after 6 h. The empty pEGFP-C1 vector was used as control. At approximately 36 h post-transfection, the cells were lysed and analyzed using real-time PCR and western blotting.

### Total RNA extraction and real-time PCR

Total RNA was extracted from the cultured cells, tumor tissues, and adjacent tissues using RNAiso Plus (Takara, Dalian, China). For each sample, 500 ng of total RNA was reverse transcribed using PrimeScript RT Master Mix (Takara), following the manufacturer’s instructions. The cDNA was subjected to real-time PCR analysis in a Rotor-gene 3000 (Corbett Research) using SYBR Premix Ex Taq II (Tli RNaseH Plus) (Takara). The cycling conditions were as follows: 95 °C for 30 s, followed by 40 cycles at 95 °C for 5 s and 60 °C for 30 s. The primer sequences were designed and synthesized by Takara. All of the primer sequences are presented in Table 2. For each sample, three replicates were analyzed. Relative mRNA expression levels were calculated using the 2^-ΔΔCt^ method, with the Ct values normalized using GAPDH as the internal control.

**Table 2:** Primers used for real-time PCR

Primers	Forward	Reverse
β2M	5′-CGGGCATTCCTGAAGCTGA-3’	5′-GGATGGATGAAACCCAGACACATAG-3’
HIF-1α	5′-GAAGTGTACCCTAACTAGCCGAGGA-3’	5′-TGAATGTGGCCTGTGCAGTG-3’
VEGF	5′-TGCCATCCAATCGAGACCCTG-3’	5′-GGTGATGTTGGACTCCTCAGTG-3’
Bcl-2	5′-CCTGTGGATGACTGAGTACCTGAAC-3’	5′-CAGAGTCTTCAGAGACAGCCAGGA-3’
HER2	5′-GACGCCTGATGGGTTAATGAG-3’	5′-GTGCTGGAGGTAGAGTGGTGAA-3’
GAPDH	5′-GCACCGTCAAGGCTGAGAAC-3’	5′-TGGTGAAGACGCCAGTGGA-3’

The primer sequence of VEGF is based on Reference [40]

### Western blotting

Cells were trypsinised and centrifuged. The pellets were then resuspended in cell lysis buffer that contained phenylmethylsulfonyl fluoride, a protease inhibitor, and a phosphatase inhibitor (Sangon Biotech, Shanghai, China). Approximately 40 μg proteins per lane were separated in 12% SDS-PAGE gels and transferred onto nitrocellulose membranes. After blocking with TBST containing 5% nonfat milk powder, the membranes were incubated with primary antibodies against β2M (1:200 dilution; in-house antibody), HIF-1α (1:500 dilution; Abcam, Shanghai, China), CREB (1:200 dilution; Abcam), phospho-CREB (Ser133) (p-CREB, 1:2000 dilution; Abcam), VEGF (1:1000 dilution; Abcam), HER2 (1:1000 dilution; Santa Cruz Biotechnology, Shanghai, China), ERK1/2 (1:100 dilution; Santa Cruz Biotechnology), phospho-ERK1/2 (Thr202/Tyr204) (p-ERK1/2, 1:200 dilution; Santa Cruz Biotechnology), serum and glucocorticoid-regulated kinase 1 (SGK1, 1:200 dilution; Bioss, Beijing, China), phospho-SGK1 (Thr256) (p-SGK1, 1:200 dilution; Bioss), and Bcl-2 (1:2000 dilution; Abcam) at 4 °C overnight. After washing with TBST, the membranes were incubated with the corresponding secondary antibodies, which were conjugated with horseradish peroxidase (Santa Cruz Biotechnology). Immunoreactive bands were visualized with SuperSignal West Pico chemiluminescent substrate (Thermo Fisher Scientific) and recorded with a ChemiDoc™ XRS+ (Bio-Rad). β-actin was used as internal control. Signal intensities were subsequently quantified using Image Lab quantification software (Bio-Rad, Hercules, CA, USA).

### IHC

Formalin-fixed, paraffin-embedded tissue specimens were obtained and handled by standard surgical oncology procedures. Serial 4-μm sections were prepared and stained using biotin-streptavidin HRP detection systems (ZSGB-BIO, Beijing, China) with the following primary antibodies: β2M (1:400 dilution; in-house antibody), HIF-1α (1:200 dilution; Bioss), VEGF (1:200 dilution; Bioss), p-CREB (1:150 dilution; ImmunoWay, Suzhou, China), p-ERK1/2 (1:100 dilution; Bioworld technology, Nanjing, China), p-SGK1 (1:200 dilution; Bioss), and Bcl-2 (1:200 dilution; Bioss). Immune complexes were visualized using a 3,3′-diaminobenzidine tetrahydrochloride substrate solution (ZSGB-BIO). The slides were then counterstained with hematoxylin and mounted. Controls were prepared by omitting the primary antibodies.

IHC staining was evaluated independently by two authors with reference to both the staining intensity and the extent of positively stained area. Staining intensity was scored as follows: 0 (negative), 1 (weak), 2(moderate), or 3 (strong). Extent of staining was scored as 0 (0%), 1 (1–25%), 2 (26–50%), 3 (51–75%), or 4 (76–100%) according to the percentages of the positively staining areas in relation to the whole carcinoma area. The sum of the intensity and extent scores was used as the immunohistochemical score (IHS), which ranged from 0 to 7. For the purpose of statistical analysis, tumors having a final staining score of ≥3 were considered positive [25].

### Statistical analysis

Statistical comparisons were performed using SPSS version 23.0. Data of the silencing and overexpression experiments were presented as the mean ± SD and analyzed by the independent-samples t test. Wilcoxon matched-pair signed-rank test was used to analyze differences in mRNA and protein expression of signaling molecules between breast cancer tissues and their matched adjacent tissues. Spearman correlation analysis was conducted to examine the strength of relationship between protein expression. *p* < 0.05 (*) or *p* < 0.01 (**) were considered statistically significant.

## Results

### Effects of β2M silencing in two types of HER2^−^ breast cancer cell lines

**Fig. 1:**
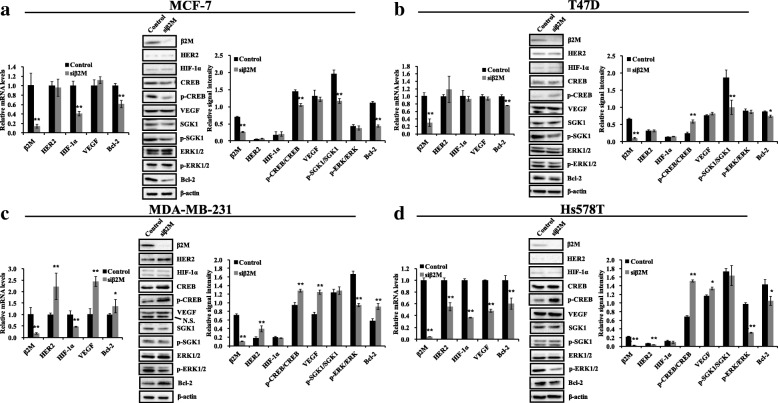
Effects of β2M silencing in two types of HER2^−^ breast cancer cell lines. Cells (MCF-7 **(a)**, T47D **(b)**, MDA-MB-231 **(c)**, and Hs578T **(d)**) were transfected with the siβ2M or control siRNA for 48 h. Total RNAs were extracted and β2M, HER2, HIF-1α, VEGF, and Bcl-2 mRNA levels were evaluated using real-time PCR. The relative mRNA levels were normalized to that of GAPDH and shown as a histogram (left of panels **a**-**d**). Whole cell lysates were prepared from cells and analyzed by western blotting for the indicated proteins. Representative immunoblots are shown in the middle of panels **a-d**. β-actin was used as a loading control. The relative protein signal intensity was quantitatively analyzed using Image Lab software and shown as histograms (right of panels **a-d**). Values were presented as the mean ± SD; * *p* < 0.05, ** *p* < 0.01 compared to the control group. N.S. indicates a non-specific band

To determine the regulatory effects of β2M silencing between ER^+^ and ER^−^ breast cancer cell lines with HER2^−^, the *β2M* gene was silenced, and possible relevant signaling molecules were analyzed by real-time PCR and western blotting. The ER^+^ and ER^−^ cells were transiently transfected with siβ2M which had a significant effect on downstream genes in our previous study [3] or control siRNA. Approximately 48 h post-transfection, real-time PCR analysis showed that the mRNA levels of β2M decreased by 85.8% (MCF-7), 71% (T47D), 82.6% (MAD-MB-231), and 96% (Hs578T), respectively (*p* < 0.01; left panels of Fig. 1a-d). The western blotting results of the β2M protein in whole cell lysates also demonstrated that siβ2M significantly reduced β2M expression compared to the control groups (*p* < 0.01; middle and right panels of Fig. 1a-d). Figure 1a shows that the siβ2M significantly reduced HIF-1α and Bcl-2 mRNA levels (*p* < 0.01), but had no effects on the mRNA levels of HER2 and VEGF (*p* > 0.05) in ER^+^ MCF-7 cells. At the protein levels, p-CREB/CREB, p-SGK1/SGK1, and Bcl-2 were significantly reduced (*p* < 0.01), whereas those of HER2, HIF-1α, VEGF, and p-ERK/ERK did not change (*p* > 0.05). In the T47D cells (Fig. 1b), which represent another ER^+^ cell line, HER2, VEGF, p-SGK1/SGK1, p-ERK/ERK, and Bcl-2 presented a similar expression profile. However, unlike the MCF-7 cells, both the mRNA and protein levels of HIF-1α did not change (*p* > 0.05), whereas p-CREB/CREB significantly increased following β2M silencing (*p* < 0.01). These results suggest that β2M silencing downregulated p-SGK1/SGK1 levels and Bcl-2 expression, but did not affect the HER2, HIF-1α, VEGF and ERK signaling in ER^+^ breast cancer cells with HER2^−^. Additionally, changes in p-CREB/CREB levels were discordant following β2M silencing.

In ER^−^ MDA-MB-231 cells (Fig. 1c), siβ2M increased the mRNA levels of HER2 (*p* = 0.007), VEGF (*p* = 0.000) and Bcl-2 (*p* = 0.040) and significantly reduced HIF-1α mRNA level (*p* = 0.001). In terms of protein expression levels, HER2, p-CREB/CREB, VEGF, and Bcl-2 were significantly enhanced (*p* < 0.01), whereas HIF-1α and p-SGK1/SGK1 did not change (*p* > 0.05), and p-ERK/ERK was significantly reduced following β2M knockdown (*p* < 0.01). In the ER^−^ Hs578T cells, the changes in the expression of HIF-1α, p-CREB/CREB, p-SGK1/SGK1, and p-ERK/ERK were similar to those in siβ2M-transfected MDA-MB-231 cells, whereas siβ2M imparted opposite effects on the expression of HER2 and Bcl-2 (Fig. 1d). Additionally, although siβ2M upregulated the protein expression level of VEGF, its mRNA expression level was downregulated in Hs578T cells (Fig. 1d). These results suggest that β2M silencing upregulates p-CREB/CREB and VEGF protein and significantly downregulates p-ERK/ERK levels, but does not affect HIF-1α protein and p-SGK1/SGK1 levels in ER^−^ breast cancer cells with HER2^−^. Additionally, changes in HER2 and Bcl-2 levels were discordant following β2M silencing.

### Effects of β2M overexpression in two types of HER2^−^ breast cancer cell lines

**Fig. 2:**
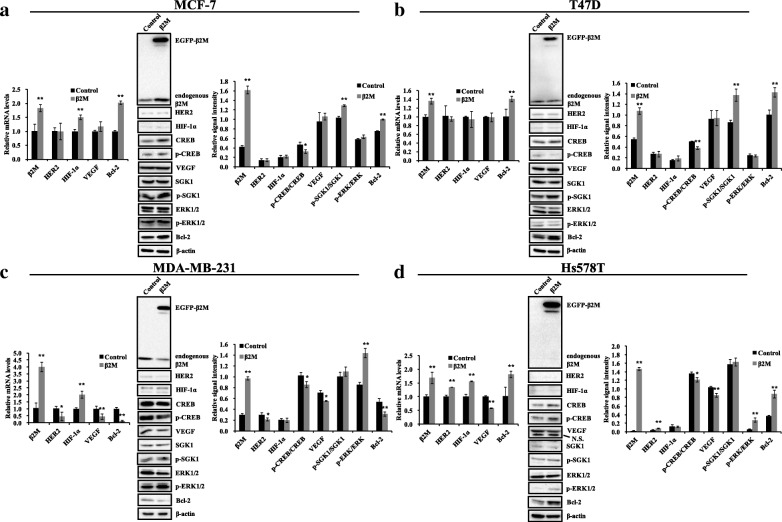
Effects of β2M overexpression in two types of HER2^−^ breast cancer cell lines. pEGFP-C1 (control) or pEGFP-C1-β2M (β2M overexpression group) plasmids were transfected into cells (MCF-7 (**a**), T47D (**b**), MDA-MB-231 (**c**), and Hs578T (**d**)) for 36 h. Total RNAs were extracted, and the mRNA levels mentioned above were evaluated using real-time PCR. The relative mRNA levels were normalized to that of GAPDH and shown as a histogram (left of panels **a**-**d**). Meanwhile, whole cell lysates were prepared from cells and analyzed by western blotting for the indicated proteins. Representative immunoblots are shown in the middle of panels **a**-**d**. β-actin was used as loading control. Relative protein signal intensities were quantitatively analyzed using Image Lab software and shown as histograms (right of panels **a**-**d**). Values are presented as the mean ± SD; * *p* < 0.05, ** *p* < 0.01 compared to the control group. N.S. indicates a non-specific band

To investigate the regulatory effects of β2M overexpression between ER^+^ and ER^−^ breast cancer with HER2^−^, we transfected cells with pEGFP-C1-β2M (β2M overexpression group) or pEGFP-C1 (control) for 36 h, and then analyzed the possible relevant signaling molecules described above by real-time PCR and western blotting. Figure 2 shows that the mRNA levels of β2M significantly increased by ~ 1.8-fold (MCF-7), ~ 1.4-fold (T47D), ~ 4-fold (MDA-MB-231), and ~ 1.7-fold (Hs578T), respectively (*p* < 0.01; left panels of Fig. 2a-d). To further determine whether β2M protein was overexpressed, we next detected the fusion protein (EGFP-β2M; ~ 39 KDa) of EGFP (27 KDa) and β2M (11.8 KDa) using the β2M antibody by western blotting. The results showed that EGFP-β2M was expressed, and its levels were significantly higher than the endogenous levels of β2M (*p* < 0.01; middle and right panels of Fig. 2a-d). In ER^+^ MCF-7 cells (Fig. 2a), real-time PCR showed that β2M overexpression had no effects on the mRNA levels of HER2 and VEGF (*p* > 0.05) and significantly increased the mRNA levels of HIF-1α and Bcl-2 (*p* < 0.01). Western blotting analysis revealed that there were no significant differences in the expression levels of HER2, HIF-1α, VEGF, and p-ERK/ERK (*p* > 0.05), p-SGK1/SGK1 and Bcl-2 were markedly upregulated (*p* < 0.01), and p-CREB/CREB was downregulated (*p* < 0.05) following β2M overexpression. In ER^+^ T47D cells (Fig. 2b), β2M overexpression resulted in a similar variation in HER2, p-CREB/CREB, VEGF, p-SGK1/SGK1, p-ERK/ERK, and Bcl-2. However, unlike MCF-7 cells, β2M overexpression had no effects on the mRNA and protein levels of HIF-1α in T47D cells. These results suggest that β2M overexpression downregulates p-CREB/CREB levels and significantly upregulates p-SGK1/SGK1 and Bcl-2 levels, but does not affect HER2, HIF-1α, VEGF, and ERK signaling in ER^+^ breast cancer cells with HER2^−^.

In ER^−^ MDA-MB-231 cells (Fig. 2c), β2M overexpression reduced the mRNA levels of HER2 (*p* = 0.017), VEGF (*p* = 0.003), and Bcl2 (*p* = 0.000) and significantly increased HIF-1α mRNA levels (*p* = 0.001). Western blotting analysis also showed that β2M overexpression downregulated HER2 (*p* = 0.043), p-CREB/CREB (*p* = 0.023), VEGF (*p* = 0.02), and Bcl-2 (*p* = 0.006), significantly upregulated p-ERK/ERK (*p* < 0.01), and did not affect HIF-1α and p-SGK1/SGK1 (*p* > 0.05). Consistent with the findings in MDA-MB-231 cells, HIF-1α, p-CREB/CREB, VEGF, p-SGK1/SGK1, and p-ERK/ERK presented similar variations in β2M-overexpressed Hs578T cells. However, β2M overexpression imparted the opposite effects on the expression of HER2 and Bcl-2 in the Hs578T cells. These results suggest that β2M overexpression downregulates p-CREB/CREB and VEGF levels and significantly upregulates p-ERK/ERK levels, but does not affect HIF-1α protein and p-SGK1/SGK1 levels in the cell lines of ER^−^ breast cancer with HER2^−^. Additionally, changes in HER2 and Bcl-2 levels were discordant following β2M overexpression.

### The correlation between β2M and the signaling molecules in ER^+^ and ER^−^ breast cancer tissues with HER2^−^

**Fig. 3:**
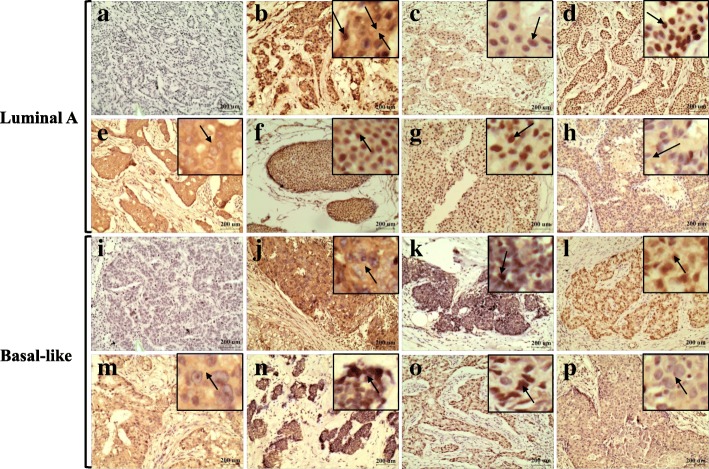
Representative immunohistochemical staining images in both HER2^−^ breast cancer tissues. **a**, **i** Cancer tissues staining without primary antibody as negative control. **b**, **j** β2M immunoreactivity. **c**, **k** HIF-1α immunoreactivity. **d**, **l** p-CREB immunoreactivity. **e**, **m** VEGF immunoreactivity. **f**, **n** p-SGK1 immunoreactivity. **g**, **o** p-ERK1/2 immunoreactivity. **h**, **p** Bcl-2 immunoreactivity. Magnification, 100×. Black boxes, a magnification of the tumor areas. Arrows, positive staining. Scale bars, 200 μm

**Fig. 4:**
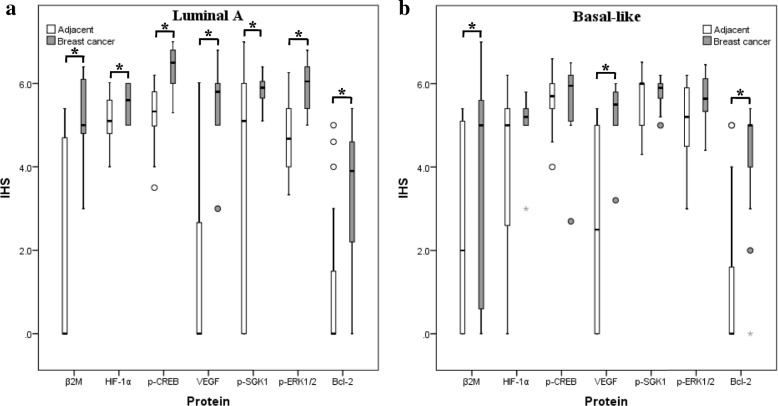
Box plots of protein expression in breast cancer and adjacent tissues. **a** Patients with luminal A breast cancer. **b** Patients with basal-like breast cancer. The length of box represents the interquartile range and the line inside represents the median.* *p* < 0.05

To further validate the results from the two types of breast cancer cell lines, the expression of β2M and signaling molecules such as HER2, HIF-1α, p-CREB, VEGF, p-ERK, p-SGK1, and Bcl-2 in the two types of HER2^−^ breast cancer tissues were examined by real-time PCR and IHC. Real-time PCR analysis did not reveal any significant differences in the mRNA levels of β2M, HER2, HIF-1α, VEGF, and Bcl-2 between the cancer tissues and adjacent tissues (*p* > 0.05; Table 3). The results of IHC are presented in Fig. 3. β2M was expressed in the cytoplasm and membrane, and nuclear staining was occasionally observed. HIF-1α, p-CREB, p-SGK1, and p-ERK1/2 were expressed in the nucleus. VEGF and Bcl-2 were expressed in the cytoplasm. The expression levels of β2M, HIF-1α, p-CREB, VEGF, p-SGK1, p-ERK1/2, and Bcl-2 were significantly higher in cancer tissues of patients with luminal A breast cancer compared to adjacent tissues (*p* < 0.05; Fig. 4a and Table 4). The expression of β2M, VEGF, and Bcl-2 were significantly higher in cancer tissues of patients with basal-like breast cancer (*p* < 0.05), and no significant differences in expression of HIF-1α, p-CREB, p-SGK1, and p-ERK1/2 were detected between cancer tissues and their matched adjacent tissues (*p* > 0.05; Fig. 4b and Table 4).

**Table 3:** The mRNA expression of β2M, HER2, HIF-1α, VEGF and Bcl-2 in two types of breast cancer tissues

	Luminal A (*n* = 29)			Basal-like (*n* = 9)		
	Fold-change median (range)	Z	p	Fold-change median (range)	Z	p
β2M	0.9202 (0.18–7.29)	− 0.622	0.534	0.6395 (0.25–2.32)	− 0.652	0.515
HER2	1.0755 (0.20–5.00)	−1.314	0.189	0.7955 (0.01–8.31)	− 0.889	0.374
HIF-1α	1.0281 (0.05–6.43)	−0.400	0.689	0.7346 (0.21–2.34)	−0.652	0.515
VEGF	0.9526 (0.19–7.86)	−0.119	0.905	1.0070 (0.02–3.59)	−0.296	0.767
Bcl-2	1.1154 (0.11–9.61)	−1.571	0.116	0.3015 (0.04–3.10)	−0.652	0.515

n The number of patients with breast cancer

**Table 4:** Comparison of protein expression between breast cancer tissues and their matched adjacent tissues

	Luminal A (*n* = 81)				Basal-like (*n* = 40)			
	Adjacent IHS median (range)	Breast cancer IHS median (range)	Z	p	Adjacent IHS median (rang)	Breast cancer IHS median (range)	Z	p
β2M	0 (0–5.4)	5 (3–6.4)	− 2.847	0.004	2 (0–5.4)	5 (0–7)	− 2.090	0.037
HIF-1α	5.1 (4–6)	5.6 (5–6)	− 2.160	0.031	5 (0–6.2)	5.2 (3–5.8)	−1.336	0.181
p-CREB	5.33 (3.5–6.2)	6.5 (5.3–7)	− 3.434	0.001	5.7 (4–6.6)	5.95 (2.7–6.5)	−0.345	0.730
VEGF	0 (0–6)	5.8 (3–6.8)	− 3.469	0.001	2.5 (0–5.4)	5.5 (3.2–6.0)	−2.867	0.004
p-SGK1	5.1 (0–7)	5.9 (5.1–6.4)	−2.272	0.023	6 (4.3–6.5)	5.9 (5–6.2)	−0.677	0.498
p-ERK1/2	4.55 (0–6.3)	6.1 (5–6.8)	−2.858	0.004	5.2 (3–6.2)	5.64 (4.4–6.5)	−1.098	0.272
Bcl-2	0 (0–5)	3.9 (0–5.4)	−2.483	0.013	0 (0–5)	5 (0–5.4)	−3.083	0.002

n The number of patients with breast cancer

Correlation analysis showed that β2M had a positive correlation with p-CREB (*r* = 0.250, *p* = 0.043), p-SGK1 (*r* = 0.310, *p* = 0.011), and Bcl-2 (*r* = 0.326, *p* = 0.007) and had no correlation with HIF-1α, VEGF, and p-ERK1/2 (*p* > 0.05) in cancer tissues of patients with luminal A breast cancer. Furthermore, a significant positive correlation was observed between p-SGK1 and Bcl-2 (*r* = 0.371, *p* = 0.001) (Table 5). However, β2M expression had no significant correlation with HIF-1α, p-CREB, VEGF, p-SGK1, p-ERK1/2, and Bcl-2 (*p* > 0.05), except that VEGF showed a strong positive correlation with Bcl-2 (*r* = 0.599, *p* = 0.000) in cancer tissues of patients with basal-like breast cancer (Table 5).

**Table 5:** Correlation between protein expression in two types of HER2^−^ breast cancer tissues

	Luminal A (*n* = 81)				Basal-like (*n* = 40)			
	β2M		Bcl-2		β2M		Bcl-2	
	r	p	r	p	r	p	r	p
HIF-1α	0.179	0.151			0.049	0.776		
p-CREB	0.250	0.043			0.250	0.141		
VEGF	0.034	0.787			0.286	0.091	0.599	0.000
p-SGK1	0.310	0.011	0.371	0.001	−0.038	0.825		
p-ERK1/2	−0.088	0.485			0.123	0.476		
Bcl-2	0.326	0.007			0.262	0.123		

n The number of patients with breast cancer

## Discussion

Breast cancer is the second most common type of cancer and a remarkably heterogeneous disease, and it is divided into four types based on the expression of ER, PR, and HER2 [24]. Each type of breast cancer has very complex molecular characteristics. ER^+^ and ER^−^ breast cancers differ in the expression of thousands of genes and show distinct patterns of mutations and alterations in the DNA copy number [26–28]. Prognosis and chemotherapy response of patients with ER^+^ and ER^−^ breast cancer are associated with different biological processes [29]. β2M has been reported as a growth-, angiogenesis-, EMT-, and bone metastasis-stimulating factor in various solid tumor malignancies [9], but inconsistent effects on breast cancer have been reported [14, 16–20]. Our previous study found that β2M expression demonstrated a significant difference in four types of breast cancer. Its protein expression was significantly associated with ER expression in breast cancer tissues, and it had distinct regulatory effects on HER2 and Bcl-2 expression in ER^+^ PR^+^ HER2^−^ and ER^−^ PR^−^ HER2^−^ cell lines [3]. However, the molecular regulatory mechanism of β2M on ER^+^ and ER^−^ breast cancers with HER2^−^ is poorly understood.

In this study, our results demonstrated the following. First, β2M activates SGK1 signaling and upregulates Bcl-2 expression, and do not affect HER2, HIF-1α, VEGF, and ERK signaling in ER^+^ breast cancer cells with HER2^−^. A previous study has reported that SGK1 signaling promotes cell survival, and SGK1 activation is dependent on the activation of PI3K and the production of PtdIns (3,4,5) P3, which could induce phosphorylation of SGK1 at its hydrophobic motif (Ser422) that in turn promotes the interaction of SGK1 with phosphoinositide-dependent kinase 1 (PDK1). Then, PDK1 activates SGK1 by phosphorylating the T-loop residue (Thr256) [30]. Subsequently, a model suggested that SGK1-Ser422 phosphorylation requires mTOR complex 1 (mTORC1) activity in ER^+^ MCF-7 and T47D cells [31]. Therefore, β2M may promote the survival of tumor cells through the mTORC1/SGK1/Bcl-2 signaling pathway in ER^+^ breast cancer cells with HER2^−^ (MCF-7 and T47D). Second, β2M inhibits CREB signaling and the expression of VEGF protein and activates ERK signaling, but does not affect HIF-1α and SGK1 signaling in ER^−^ breast cancer cells with HER2^−^. CREB, a transcription factor, is involved in the tumorigenicity of HER2/Neu-overexpressing tumor cells [32, 33]; VEGF, a CREB target gene, is associated with tumor angiogenesis, metastatic growth, and poor prognosis in breast cancer [34–36]. Activation of ERK survival signaling can be triggered by β2M treatment [11] or overexpression [12] in human renal cell carcinoma SN12C cells. Therefore, β2M may inhibit the survival of tumor cells through the inhibition of CREB/VEGF signaling, and promote the survival of tumor cells through the activation of ERK signaling in ER^−^ breast cancer cells with HER2^−^. The reason behind the observed opposite regulatory effects of β2M on ER^−^ breast cancer cells with HER2^−^ may be that the triple-negative breast cancer is a special type of breast cancer that has special biological behavior and a very complex regulatory mechanism. Third, β2M exhibits different regulatory effects on CREB and p-CREB in the cell lines of ER^+^ breast cancer with HER2^−^ (MCF-7 and T47D), and HER2 and Bcl-2 in the cell lines of ER^−^ breast cancer with HER2^−^ (MDA-MB-231 and Hs578T), which may be due to the fact that breast cancer has a very complex molecular regulatory mechanism, and every type of breast cancer has several subtypes, and these signaling molecules may be regulated by other pathways in corresponding subtypes of breast cancer. Fourth, β2M is positively correlated with p-CREB, p-SGK1, and Bcl-2 and has no correlation with HIF-1α, VEGF and p-ERK1/2, and p-SGK1 has a significantly positive correlation with Bcl-2 in cancer tissues of patients with luminal A breast cancer, which agrees with the results obtained from the same molecular types of breast cancer cells except CREB signaling. However, β2M expression shows no significant correlation with HIF-1α, p-CREB, VEGF, p-SGK1, p-ERK1/2, and Bcl-2 except for VEGF, which shows a strong positive correlation with Bcl-2 in cancer tissues of patients with basal-like breast cancer, thereby showing discordance with the results obtained from the same molecular types of breast cancer cells. One possible reason is that large differences may exist among cancer cell lines and tissue samples, particularly in terms of its molecular genome [37, 38], and cell lines only mirror some but not all of the molecular properties of primary tumors [24]. In addition, VEGF protein is upregulated, whereas VEGF mRNA is downregulated following β2M silencing in Hs578T cells. Previous study has also shown that some proteins are negatively correlated with the mRNA expression in lung adenocarcinomas, which may reflect negative feedback on the mRNA or the protein or the presence of other regulatory influences [39]. Therefore, VEGF protein may have a negative feedback on VEGF mRNA, resulting in the rapid degradation and downregulation of the mRNA when increasing to the peak following β2M silencing. Consequently, β2M may promote tumor survival through the SGK1/Bcl-2 signaling pathway in ER^+^ breast cancer with HER2^−^ and have no regulatory effects in ER^−^ breast cancer with HER2^−^. It is possible that the regulatory function of β2M is correlated with ER expression in HER2^−^ breast cancer. The regulatory mechanism of β2M in HER2^−^ breast cancer needs to be further investigated.

## Conclusions

β2M has different molecular regulatory mechanisms between ER^+^ and ER^−^ breast cancer with HER2^−^. β2M may promote tumor survival through the SGK1/Bcl-2 signaling pathway in ER^+^ breast cancer with HER2^−^ and have no regulatory effects on ER^−^ breast cancer with HER2^−^. Understanding the regulation of β2M-mediated signaling pathways will help to identify novel therapeutic targets for patients with different types of breast cancer.

## Abbreviations

Bcl-2: B-cell lymphoma 2
CREB: cAMP-responsive element binding protein
DMEM: Dulbecco’s modified Eagle’s medium
EMT: epithelial to mesenchymal transition
ER: estrogen receptor
ERK: extracellular signal-regulated kinase
FBS: fetal bovine serum
HER2: human epidermal growth factor receptor 2
HFE: hemochromatosis protein
HIF-1α: hypoxia-inducible factor-1α
HLA: human leukocyte antigen
IHC: immunohistochemistry
IHS: immunohistochemical score
mTOR: mammalian target of rapamycin
mTORC1: mTOR complex 1
p-CREB: phospho-CREB (Ser133)
PDK1: phosphoinositide-dependent kinase 1
p-ERK1/2: phospho-ERK1/2 (Thr202/Tyr204)
PI3K: phosphatidylinositol 3-kinase
PKA: protein kinase A
PR: progesterone receptor
p-SGK1: phospho-SGK1 (Thr256)
RT-PCR: reverse transcription-PCR
SGK1: serum and glucocorticoid-regulated kinase 1
siRNA: small interfering RNA
siβ2M: β2M siRNA
VEGF: vascular endothelial growth factor
VEGFR-2: vascular endothelial growth factor receptor-2
β2M: β2-microglobulin
